# Breast Implant Illness: An Expert-Panel Discussion on Current Research

**DOI:** 10.1093/asjof/ojab027

**Published:** 2021-06-23

**Authors:** Jeffrey M Kenkel, Caroline Glicksman, Patricia McGuire, Luis Rios, William P Adams

**Affiliations:** 1 Department of Plastic Surgery, UT Southwestern Medical Center, Dallas, TX, USA; 2 Hackensack Meridian School of Medicine at Seton Hall, Nutley, NJ, USA; 3 Washington University, St. Louis, MO, USA

The authors are pleased to present an expert roundtable discussion on breast implant illness (BII) (Video). The discussants include a panel of leading experts in aesthetic surgery, and the roundtable was recorded live at The Aesthetic Meeting 2021 in Miami Beach, FL, USA. The panelists provide an overview of the current challenges, areas of research, and education being undertaken to better understand the symptoms of BII and how they present in various patient populations. They discuss not only the physical indications of BII but also the emotional and psychological implications that confound its diagnosis and treatment. Finally, the panelists discuss the contributions of The Aesthetic Society and the Aesthetic Surgery Education and Research Foundation (ASERF) toward improving the education of BII to restore health and balance and improve patient outcomes.

